# Evaluation of Xerostomia and salivary flow rate in Hashimoto’s Thyroiditis

**DOI:** 10.4317/medoral.20559

**Published:** 2015-11-22

**Authors:** Farzaneh Agha-Hosseini, Nooshin Shirzad, Mahdieh-Sadat Moosavi

**Affiliations:** 1 DDS, MSc, Fellowship of Research of Biology. Professor, Dental Research Center / Department of Oral Medicine, School of Dentistry, Tehran University of Medical Sciences, The Academy of Medical Sciences, Tehran, Iran; 2 Assistant Professor, Endocrinology and Metabolism Research Center, Endocrinology and Metabolism Clinical Sciences Institute, Endocrinology and Metabolism Research Institute, Tehran University of Medical Sciences, Tehran, Iran; 3 DDS, MSc. Assistant Professor, Dental Research Center / Department of Oral Medicine, School of Dentistry, Tehran University of Medical Sciences, Tehran, Iran

## Abstract

**Background:**

One of the most common causes of hypothyroidism is Hashimoto´s Thyroiditis (HT). Early detection of dry mouth is critical in preserving and promoting systemic and oral health. In this study we have assessed, for the first time, salivary function and xerostomia in HT patients who have not been involved with Sjögren´s syndrome.

**Material and Methods:**

HT was diagnosed in 40 patients based on clinical findings and positive anti-thyroid peroxidase antibodies (anti-TPO). Controls, matched by sex, age and body mass index (BMI), and with no history of thyroid disease, were selected. A questionnaire was used for diagnosis of xerostomia. Saliva samples were taken between 8 a.m. and 9 a.m., and at least 2 hours after the last intake of food or drink. The flow rate was calculated in milliliters per minute.

**Results:**

Xerostomia was significantly higher in patients with HT. Unstimulated salivary flow rate was significantly lower in the HT group. Stimulated salivary flow rate was lower in HT group, but the difference was not significant.

**Conclusions:**

The patients with HT experienced xerostomia, and their salivary flow rate was diminished. Spitting the saliva then assessing salivary flow rate based on milliliter per minute is non-invasive, fast, and simple for chair-side diagnosis of dry mouth. Autoimmune diseases can be accompanied by salivary gland dysfunction. This may be due to the effect of cytokines in the autoimmune process or because of thyroid hormone dysfunctions.

**Key words:**Thyroid, salivary gland, xerostomia.

## Introduction

One of the most common causes of hypothyroidism is Hashimoto’s Thyroiditis (HT), an autoimmune disease of the thyroid gland and a typical example of an organ-specific autoimmune disorder (1,2).

Xerostomia and decreased salivary flow rate in HT is expected, even without association with Sjogren’s syndrome, for the following reasons: Sjogren’s syndrome and autoimmune thyroiditis, have many genetic and immunopathological similarities. They have some features in common such as both are equally more common in females than in males, and the peak of prevalence occurs between 30 and 50 years (3). Their immunopathological similarities include autoimmunity in epithelial cells, T lymphocytic infiltration, epithelial HLA class II molecules expression, clonally B expansion and the probability of developing mucosa-associated lymphoid tissue (MALT) lymphoma (3).

There are articles which credit salivary changes as a mirror of the body, reflecting endocrine and other biological changes in patients with xerostomia (4,5), and one of the essential components in maintaining good oral hygiene is proper qualitative and quantitative saliva (6).

Oral hygiene has significant association with various diseases such as cardiovascular disease and airway infection. Periodontal disease has been associated with the onset, progression, and severity of autoimmune disorders. As cardiovascular disease and airway infection may increase morbidity in patients with autoimmune disease, good oral hygiene may have an important, positive influence (6).

The aim of this study is the evaluation of salivary function and xerostomia in Hashimoto patients who have not suf-fered from Sjögren´s syndrome.

## Material and Methods

**Figure 1:**
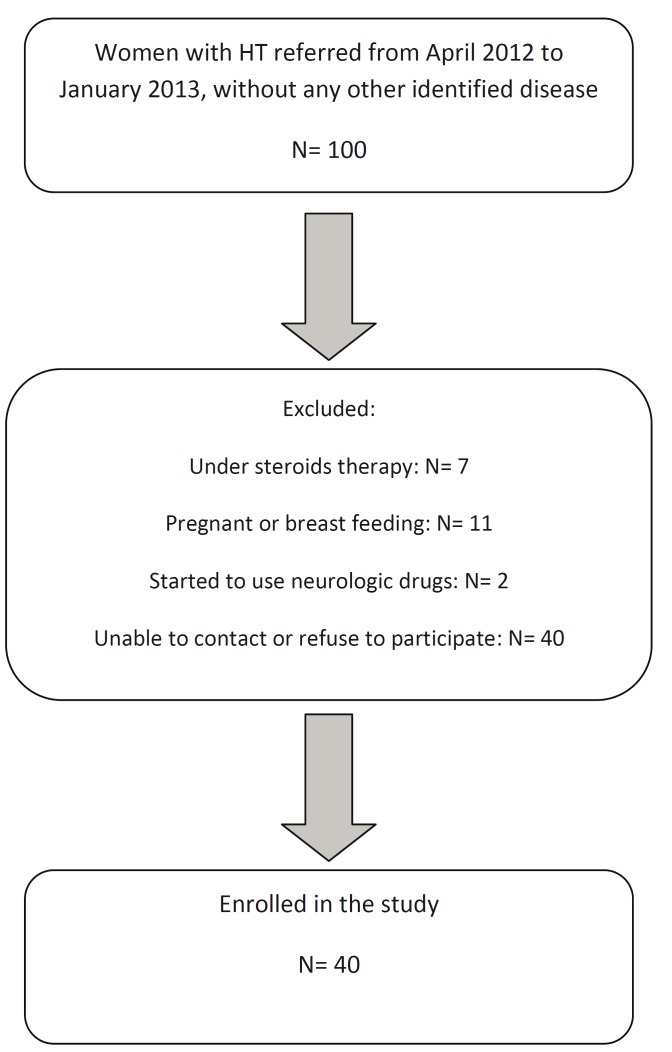
Enrolled and excluded subjects.

Forty women from the outpatient unit of Arash Hospital (Tehran University of Medical Sciences), and diagnosed with Hashimoto’s Thyroiditis, were enrolled in this study through convenience sampling (Fig. 1). Diagnosis was based on clinical findings and positive anti-thyroid peroxidase antibodies (anti-TPO). All patients were under thyroxine therapy and none of them was hypothyroid at the time of the study. Thyroxine is not a xerogenic medication.

Sex-, age-, and body mass index (BMI) frequency matched controls, with no history of thyroid disease, were selected. The controls were selected from the women staff of our department. Exclusion criteria were smoking, taking xerogenic medical agents, oral candidiasis or unfavorable oral health conditions (pocket depth more than 3 mm), history of head and neck radiation and diagnosis of an immunological disorder, diabetes, any infectious disease, any malignancy, or any other systemic disease.

Patient informed consent was obtained before commencing the study, and the protocol was approved by the Ethics Committee of Tehran University of Medical Sciences, Iran.

The women in both groups were asked to complete a questionnaire that contains a list of symptoms associated with xerostomia (7,8). A second questionnaire was used to evaluate the severity of xerostomia (9,10). Xerostomia inventory (XI) score was defined as the severity of dry mouth feeling (11). The response to each question was scored as follows: 1 = never, 2 = hardly, 3 = occasionally, 4 = fairly often and 5 = very often. The score to all 11 questions were summed for each individual. The range of XI scores was therefore 11 (11 × 1) to 55 (11 × 5).

Blood specimens were obtained from both groups to determine the presence of auto antibodies (anti-TPO, an-ti-thyroglobulin (Tg), anti-Ro/SSA, anti-La/SSB, auto antibodies to nucleus and anti-nature double-strand DNA).

We ruled out Sjogren’s syndrome in HT patients based on the 2012 American College of Rheumatology (ACR) Classification Criteria (11). Patients were assessed for two of three criteria: anti-Ro/SSA, anti-La/SSB and Keratoconjunctivitis sicca.

- Saliva collection

Stimulated and unstimulated whole-saliva samples were taken under resting conditions in a quiet room between 8 a.m. and 9 a.m., and at least 2 hours after the last intake of food or drink. Unstimulated salivary samples were obtained by expectoration without chewing movements. Subsequently, participants chewed a piece of paraffin of identical size. 60 seconds after pre-stimulation, the participants were asked to swallow the saliva present in the mouth and then samples were obtained by expectoration. Stimulated and unstimulated whole-saliva samples were collected in a calibrated and dry plastic tube. Saliva was collected over a period of 5 minutes. The flow rate was calculated in milliliters per minute (4).

- Statistical analysis

Statistical analysis was performed using SPSS version 16 software. The normal distributions of continuous variables were assessed by the Kolomogrov-Smirnov test. The continuous variables are expressed as mean ± S.D. or median (interquartile range) as appropriate, and categorical variables are expressed as a number (%). Student´s t-test was used to compare mean values between groups where the distribution was normal, and Mann-Whitney non-parametric test was used when the distribution of values was not normal.

The odds ratio (OR) of each variable and the 95% confidence intervals (CI) were calculated. The significance level (*P* value) for all of the analyses was defined as *P*<0.05. Binary logistic regression analysis was performed to analyze the predictive value of parameters.

## Results

The characteristics of case and control participants are shown in  Table 1. Subjects who had at least 3 positive re-sponses to the questionnaire, with a list of symptoms associated with xerostomia formed the xerostomic individuals (5). Women with at least 3 positive responses were selected as xerostomic subjects, because some questions (questions 2 and 9) may be related to other disorders and are not specific to xerostomia. Six women in control and 16 women in HT group had xerostomia. So xerostomia was significantly higher in patients with HT.

Mann–Whitney non parametric test showed that unstimulated salivary flow rate was significantly lower in HT group. (*P* < 0.05,  Table 2). Stimulated salivary flow rate was lower in HT group, but the difference was not significant ( Table 2).

**Table 1:**
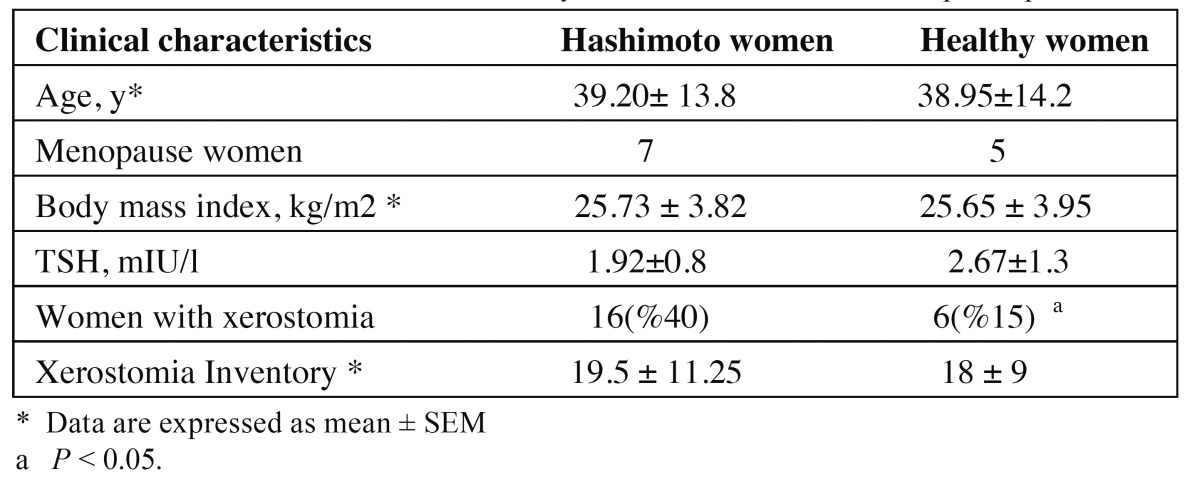
Clinical characteristics and salivary flow rate of case and control participants.

**Table 2:**
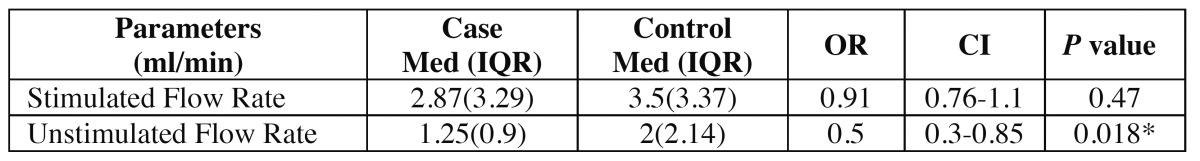
Result of stimulated and unstimulated flow rates.

The value of unstimulated salivary flow rate as a predictor for the presence of HT was studied using logistic regression analysis. The adjusted OR for probability decrease of HT for 1 ml/min increase in salivary flow rate was 50 % (OR= 0.50, 95% CI: 0.30 - 0.84).

Xerostomia Inventory was increased in HT patients but it was not significant (*P* = 0.14).

The prevalence of Anti-TPO and Anti-Tg was 100% and 40% in HT subjects, and 0% and 10% in controls respectively. The results of other antibodies are shown in  Table 3. All patients were negative for anti-Ro/SSA and anti-La/SSB antibodies, and none of them had Keratoconjunctivitis sicca symptoms or positive responses to subjective symptoms related to Sjögren´s syndrome (dryness feeling in eyes, lip and the nose - questions 9, 10 and 11 of xerostomia Inventory). According to ACR criteria, participants with rheumatoid arthritis, systemic lupus erythematosus, scleroderma, or other connective tissue disease, were excluded. Also, only one patient was positive for ANA and anti dsDNA, which rules out the superimposition of other autoimmune connective tissue disorders. In fact we have excluded this important confounding factor.

**Table 3:**
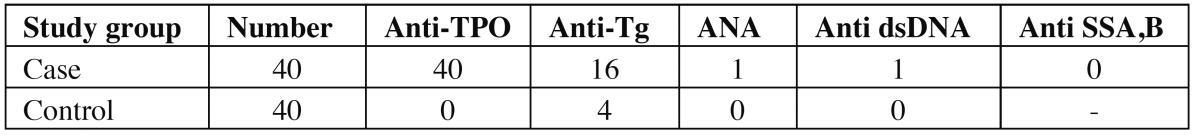
Number of subjects with positive autoantibodies in case and control.

## Discussion

HT is a frequent form of autoimmune thyroid disease, affecting up to 2% of the general population; this is twice the prevalence of type 1 diabetes (12).

In this study, in the absence of Sjogren syndrome, patients with HT experienced xerostomia and also had diminished salivary flow rate. Confounding factors such as menopause status, which is a xerogenic factor, had no effect on this result because there was no significant difference between menopausal women in the two groups. The negative impact of hypo salivation on oral hygiene in patients with autoimmune diseases certainly increases morbidity in these patients. Therefore, salivary assessment for early detection of dry mouth is essential for maintaining and promoting systemic and oral health (6).

As Szanto A and his colleagues have suggested, autoimmune changes in endocrine glands such as thyroid, may also occur in exocrine glands such the salivary gland, because the process of both is secretion (13). These changes may occur in the salivary glands of HT patients too, and may cause salivary flow rate changes.

Sjögren´s syndrome is an autoimmune exocrinopathy often associated with Hashimoto´s Thyroiditis, and the probability of this association will increase through time (3,13). Therefore one of the most important considerations for a study on salivary changes in HT is ruling out Sjögren´s syndrome in these patients.

To the best of our knowledge, there have been only two studies which assessed xerostomia and salivary flow rate in HT. Both had an identical method, and patients with autoimmune thyroiditis enrolled in the study had HT for 10 years. One of the most important limitations of those studies was the lack of Sjögren´s syndrome exclusion, especially after 10 years with HT (14,15).

For the first time, we have assessed xerostomia, and stimulated and unstimulated whole salivary flow rate, in patients with HT not suffering from Sjögren´s , based on ACR Classification Criteria (11). The diagnosis of dry mouth based on a minor salivary gland biopsy of the lips, Saxon test, sialography, and scintigraphy, requires complex manipulation, takes a long time for diagnosis, and is invasive (6). Another advantage of our study, compared to previous studies, is the technique of salivary collection. Quantitating unstimulated and stimulated whole saliva flow rates (sialometry) is the most supported clinical method for diagnosing salivary gland dysfuncti (16) Spitting the saliva then assessing salivary flow rate based on milliliter per minute is noninvasive, fast, and simple for chair-side diagnosis of dry mouth.

The exact mechanism of impaired salivary secretion in autoimmune diseases other than Sjögren´ syndrome is not well understood, and studies based on salivary gland biopsy and radiological techniques may resolve this question (6).

The proposed mechanisms involved in the inhibition of the secretory process in these patients may be related to cytokines. Previous studies introduced interferon (IFN) as an independent factor influencing salivary gland function. They have shown that in different chronic inflammatory conditions, exposure of salivary glands to IFNs and pro inflammatory cytokines such as IL6 interferes with their ability to make saliva and this functional loss was reversible and was independent from sialoadenitis or auto antibody production. A complete recovery of salivary gland function was made by the removal of innate stimuli (17).

Both immune cells and thyroid cells themselves secrete IFN-γ in HT, and it may be responsible for the continuation of the inflammatory process within the thyroid gland (18,19). So it could reasonably be proposed that IFNs are an important pathogenic factor in salivary gland hypofunction in HT.

IFNs can induce apoptosis, but the consensus from previous studies is that hypofunction is ‎not due to an increase in apoptotic cell numbers in the glands.

A suggested mechanism involved in IFN and IL-6-mediated salivary gland hypo function is exposure of salivary gland acinar cells to cytokines such as TNF-α and IFN-γ, which has been shown to disturb the integrity of tight junctions, thus causing disturbances in water transport (17). In various epithelia, cytokines are known to change epithelium integrity via alteration of junction proteins expression (19).

There are other salivary gland hypo function mechanisms in Sjögren´s syndrome, consisting of:

1. Inhibition of neurotransmitter release by pro-inflammatory cytokines such as IL-1β, IL-6 and TNF-α (all of these cytokines are known to have a role in HT pathogenesis).

2. Increased degradation of acetylcholine by cholinesterase (cholinesterase is increased in saliva and salivary glands of Sjögren´s syndrome patients).

3. Glandular hypo function by reducing the water content of saliva caused by blockage of muscarinic acetylcholine receptor M3.

4. Altered calcium signaling (19,20).

Each of these factors may contribute to hypo salivation in other autoimmune diseases such as HT, therefore further evaluations are necessary.

In a new animal study, tyrosylprotein sulfotransferase-2 knockout mice have salivary hypo function and smaller salivary gland size due to primary hypothyroidism. By thyroid hormone supplementation, all signs of hypothyroidism (serum T4 and body weight) and salivary gland hypo function (histological changes and pilocarpine-induced salivary flow) were restored to normal or near normal (21). We can conclude that in HT, the auto immunity process together with the role of thyroid hormones trophic effect on the salivary gland may induce salivary gland hypo function. In our patients, thyroxine therapy restored thyroid function and they became euthyroid; but they had xerostomia because, while salivary changes related to thyroxine function may have improved, there was no effect on possible autoimmune destruction of salivary glands. Further research may reveal if an immunosuppressant agent can alleviate salivary changes.

In the current study, stimulated salivary flow rate was not significantly diminished in HT, so salivary stimulation may relieve xerostomia in these patients. Management of xerostomia includes drug administration (systemic secreto-gogues, saliva substitutes and bile secretion stimulator), night guard, diet and habit modifications, and salivary output stimulators like chewing sugar-free, xylitol-containing mints, candies, and gum (22). One of the limitations of the current study is the limited follow-up with patients because of cross-sectional study design. We suggest further longitudinal studies to evaluate salivary changes in HT.

## Conclusion

Our data show that autoimmune diseases such as HT can be accompanied by salivary gland dysfunction, regardless of the absence of Sjögren´s syndrome. This may be due to the effect of cytokines in the autoimmune process or because of thyroid hormone dysfunctions.
